# Detection of aqueous VEGF concentrations before and after intravitreal injection of anti-VEGF antibody using low-volume sampling paper-based ELISA

**DOI:** 10.1038/srep34631

**Published:** 2016-10-11

**Authors:** Min-Yen Hsu, Yu-Chien Hung, De-Kuang Hwang, Shang-Chi Lin, Keng-Hung Lin, Chun-Yuan Wang, Hin-Yeung Choi, Yu-Ping Wang, Chao-Min Cheng

**Affiliations:** 1 Department of Ophthalmology, Taichung Veterans General Hospital, Taichung 407, Taiwan; 2 Institute of Nanoengineering and Microsystems, National Tsing Hua University, Hsinchu 300, Taiwan; 3 Rong Hsing Research Center for Translational Medicine, National Chung Hsing University, Taiwan; 4 Center for Translational Medicine, Department of Medical Research, Taichung Veterans General Hospital, Taichung 407, Taiwan; 5 Department of Ophthalmology, School of Medicine, National Yang-Ming University, Taipei, Taiwan; 6 Department of Ophthalmology, Taichung Tzu-Chi Hospital, Taichung 40, Taiwan; 7 Department of Radiology, Taichung Veterans General Hospital, Taichung 407, Taiwan; 8 Institute of Biomedical Engineering, National Tsing Hua University, Hsinchu 300, Taiwan

## Abstract

Intraocular vascular endothelial growth factor (VEGF) levels play an important role in the pathogenesis of blindness-related diseases, such as age-related macular degeneration (AMD). Here, we aimed to develop a paper-based enzyme-linked immunosorbent assay (P-ELISA) to analyze the suppression of aqueous VEGF concentrations following intravitreal injection (IVI) of anti-VEGF antibody (bevacizumab or ranibizumab). A total of 25 eyes with wet AMD, one with myopic neovascularization, and one with polypoidal choroidal vasculopathy were enrolled in this study. The limit of detection using P-ELISA was 0.03 pg/mL. Forty-six consecutive samples of aqueous humor were acquired. From all samples, 66.67% (10/15) achieved complete VEGF suppression (below the detection limit) within 5 weeks of receiving IVI of anti-VEGF antibody. Only 13.33% of samples (2/15) achieved complete VEGF suppression 5 weeks after receiving treatment. In some patients, elevated VEGF was still detected 5 weeks after receipt of anti-VEGF antibody, and all samples (10/10) were found to have elevated VEGF levels 49 days after treatment. Thus, we suggest that monthly IVI of anti-VEGF antibody may be required to ensure durable VEGF inhibition. Ultrasensitive P-ELISA can detect elevated VEGF at an earlier time point and may facilitate decision-making regarding appropriate treatment strategies.

The prevalence of age-related macular degeneration (AMD) has gradually increased in developed countries^1^,^2^. Angiogenesis within the retina plays a critical role in choroidal neovascularization (CNV) formation and causes devastating complications, such as blindness^3^,^4^. Angiogenesis results from a complex cascade of mechanisms and can be activated by several factors, including vascular endothelial growth factor (VEGF), platelet-derived growth factor (PDGF), fibroblast growth factor (FGF), transforming growth factor-alpha and -beta, angiopoietin-1, and angiopoietin-2^5^,^6^.

Within the last decade, intravitreal injection (IVI) therapy using anti-VEGF agents (e.g., aflibercept, bevacizumab, and ranibizumab) has emerged as an essential treatment strategy for tackling many forms of ocular neovascularization in AMD, polypoidal choroidal vasculopathy (PCV), and diabetic retinopathy^7^,^8^. VEGF has been proven to play a critical role in AMD, and suppression of VEGF levels within the eyeball after IVI of anti-VEGF antibody has been shown to restore or prevent further visual acuity impairment^9^. Positive correlations between aqueous humor VEGF levels and vitreous VEGF levels have been observed in patients with AMD^10^. Moreover, loss of intraocular VEGF suppression is always followed by morphological changes, as determined by spectral-domain optical coherence tomography (SD-OCT), and such changes typically and ultimately result in loss of visual acuity^9^.

Many research efforts have been undertaken to identify the pharmacodynamics of IVI of anti-VEGF antibody and to optimize injection intervals for maximum therapeutic effect^11^,^12^,^13^,^14^,^15^,^16^. However, some patients with wet AMD have shown no response, even after anti-VEGF drug injections; these patients have been termed nonresponders^17^. Notably, persistent macular edema remains evident in nonresponders, even after several months of anti-VEGF injections^18^. With quantitative and rapid testing, intraocular VEGF can be measured in outpatient clinics, and ophthalmologists can more easily measure and adequately treat even the nonresponders by shifting them to an alternate treatment protocol (e.g., different anti-VEGF drugs, anti-PDGF drugs, or photodynamic therapy) before vision loss occurs.

Under treatment strategies based on early detection and prompt treatment, point-of-care (POC) biochemical diagnostics (e.g., Luminex or conventional enzyme-linked immunosorbent assay [ELISA]) for the detection of aqueous VEGF elevation before retinal structural changes can be a powerful diagnostic test for guiding therapy^9^,^19^,^20^. The optimal interval between serial monthly or bimonthly IVI anti-VEGF injection also needs to be determined by examining true aqueous VEGF levels rather than by determining structural changes via SD-OCT^14^.

Paper-based ELISA (P-ELISA) has been shown to be a successful semiquantitative biomarker for evaluation of various diseases, such as, but not limited to, human immunodeficiency virus (HIV),^21^ dengue virus,^22^ NC16 (auto-antibody) in the bullous pemphigus,^23^ and lactoferrin on the cornea epithelium.^24^ Aqueous humor VEGF levels range from 10^−14^ to 10^−6^ g/mL^25^−^26^ and can be quantified by P-ELISA without sample dilution within one hour. One of the major benefits of P-ELISA is the ability to use very small sample volumes (e.g., only 40 μL) for each sample of aqueous VEGF.

Accordingly, in this study, we used P-ELISA as a POC diagnostic tool to quantify aqueous humor VEGF levels before and after IVI of anti-VEGF antibody.

## Material and Methods

### Patients

Patients undergoing IVI of anti-VEGF antibody (bevacizumab or ranibizumab) for AMD, PCV, or myopic neovascularization were recruited at the Department of Ophthalmology of Taichung Veterans General Hospital. Eyes previously operated on within the last 3 months were excluded. The protocols used in this study conformed to the tenets of the Declaration of Helsinki and were approved by the Institutional Review Board of Taichung Veterans General Hospital (IRB number: CF14120). Informed consent for aqueous tapping during the IVI procedure was obtained from all patients after an explanation of the study. All aqueous humor samples were collected from August 2014 to February 2015 (n = 46).

### Aqueous humor collection and IVI injection

Patients received IVI of bevacizumab (2.5 mg/0.1 mL; Avastin; Roche, Switerland) or ranibizumab (0.5 mg/0.05 mL; Lucentis; Genentech, USA). The strategy for the injection was based on an “as needed” regimen^27^,^28^,^29^. Monthly SD-OCT was performed for evaluation, and IVI of anti-VEGF antibody treatment was applied in case of reoccurrence of retinal bleeding or fluid accumulation on SD-OCT. All patients were followed up at the OPD for at least 3 months.

Immediately before each IVI, aqueous sampling was performed by aspirating 0.1 mL of aqueous humor using a 30-gauge needle connected to an insulin syringe at the temporal limbus. IVI of anti-VEGF antibody was then performed used a 30-gauge needle in the inferotemporal quadrant at 3.0 mm to 3.5 mm posterior to the limbus. The undiluted aqueous samples were stored in a −70 °C freezer until analysis.

### Measurement of VEGF

**Figure 1:**
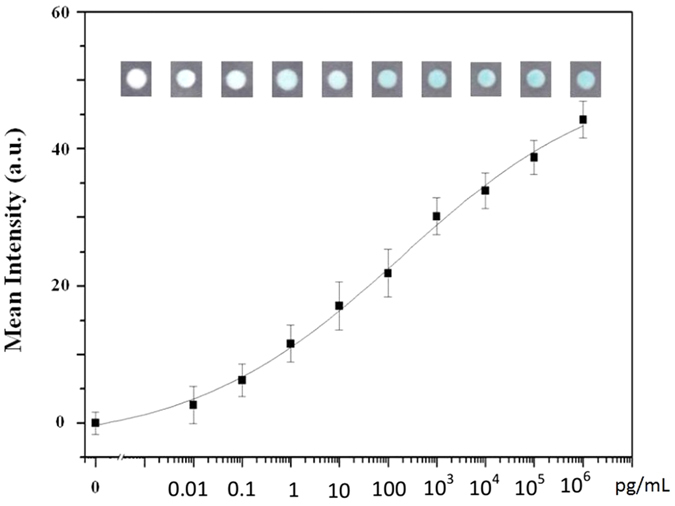
Calibration curve of paper-based ELISA for VEGF detection. We used a commercial kit for VEGF labeling at different concentrations ranging from 0.01 to 10^5 ^pg/mL.

P-ELISA uses wax to form a hydrophobic zone, and the reaction can be accomplished within a 1-cm-diameter hydrophilic zone^21^. Thus, we used horseradish peroxidase (HRP) to conjugate with bevacizumab as the antibody source to perform the colorimetric reaction, and the protocol was carried within 1 h, as previously described^25^,^26^. Briefly, the test zone was hydrated using 2 μL PBS. The residual PBS was then removed, and 2 μL VEGF protein was added as the antigen. The sample was then held for 10 min, and 2 μL BSA was added. After another 10 min, 5 μL Avastin-HRP-conjugated antibody was added and incubated for 10 min, followed by addition of 2.5 μL streptavidin. The excess antibody was then washed away, and 2 μL TMB+H_2_O_2_ (diluted 1:2) was then added for the colorimetric reaction. All procedures were carried out under a laminar flow hood in order to reduce bias, such as humidity and evaporation. We then used a smartphone camera to capture the colorimetric results every minute. After drying of the test zone, the final colorimetric results were scanned using a desktop scanner. All results were analyzed using Photoshop software. We used a commercial VEGF kit to generate a calibration curve for VEGF concentration versus the intensity of colorimetric results (Fig. 1). The correlation efficient (R^2^) of the calibration curve was 0.9938 in Hill’s equation model. The standard deviation of the intensity for the blank test was 1.6617 (n = 24), and three times the standard deviation was 4.9851, which was considered the limit of detection (LOD). After fitting back this LOD intensity into Hill’s equation, the LOD for the VEGF concentration was determined to be 0.03 pg/mL. The comparison between conventional ELISA and P-ELISA for aqueous humor VEGF detection is shown in Table 1.

### Measurement of central foveal thickness (CFT) and visual acuity

**Figure 2:**
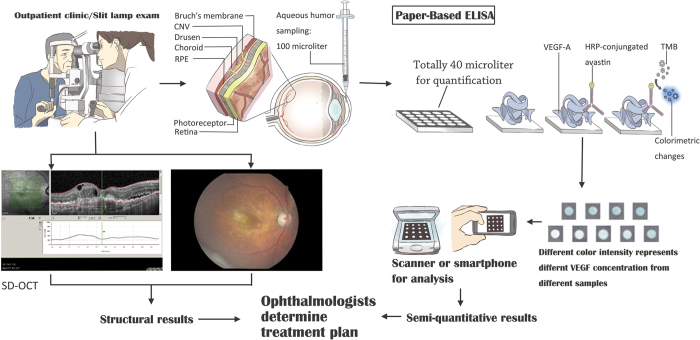
Schematic illustration of paper-based ELISA for VEGF detection. In the clinical setting, patients receive standard-of-care procedures: slit lamp, SD-OCT, and FAG (left panel). Paper-based ELISA requires 1 μL of aqueous humor before operation, and quantification of VEGF concentrations requires 40 μL (right panel). Further colorimetric results can be obtained using a scanner or smartphone. Ophthalmologists can determine treatment according to VEGF concentrations and SD-OCT results.

The CFT was measured by SD-OCT (Heidelberg Spectralis HRA+OCT) at each time point (Fig. 2). Visual acuity was measured using the Snellen chart and was converted to logMAR.

### Statistical analysis

Aqueous concentrations of VEGF and clinical data are expressed as means ± standard deviations (SDs). To evaluate the association of postoperative duration with VEGF concentrations and other clinical data described earlier, samples were divided into three groups according to the timing of IVI: group 1, before IVI; group 2, post-IVI within 5 weeks; and group 3, post-IVI more than 5 weeks. To evaluate the associations between different IVI agents and the VEGF suppression effect, samples were also divided into two groups based on agents of IVI: (1) the bevacizumab group and (2) the ranibizumab group. Wilcoxon rank-sum tests and Fisher’s tests were used to compare VEGF concentrations and other clinical data among the three groups. Additionally, Fisher’s tests were used to evaluate the associations between different IVI agents and earlier VEGF elevation. Results with *P* values of less than 0.05 were considered statistically significant. All analyses were carried out using Graphpad Prism software. From intensity analysis, data points having intensities of less than 4.9851 were considered below the LOD. In statistical analysis, VEGF concentrations of 0.015 pg/mL were considered below the LOD according to our statistical assumptions

## Results

### Basic characteristics

**Figure 3:**
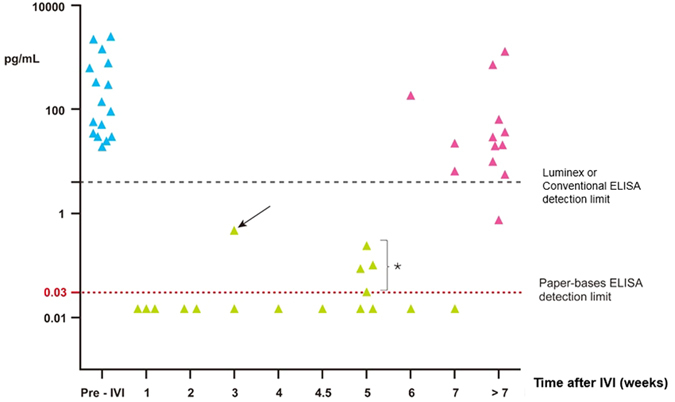
Aqueous VEGF levels before and after intravitreal injection of anti-VEGF antibody. Blue dots represent VEGF levels before IVI (group 1). Black dots represent VEGF concentrations after IVI. The horizontal red dotted line is the detection limit of paper-based ELISA, approximately 0.03 pg/mL. The horizontal black dashed line is the detection limit of conventional ELISA or Luminex, approximately 4–5 pg/mL. The earliest elevation of VEGF was found in a patient with PCV at 3 weeks after IVI (arrow). Four earlier elevations of VEGF levels were found 5 weeks after IVI injection by ultrasensitive paper-based ELISA (asterisk).

Twenty-seven consecutive eyes were enrolled prospectively in this study. Twenty-five eyes had wet AMD, one eye had myopic neovascularization, and one eye had PCV. The diagnosis of PCV was confirmed with fluorescence angiography and indocyanine green angiography (FAG/ICG). AMD or PCV was diagnosed by analysis of FAG/ICG by three retina specialists. A total of 46 aqueous humor samples were acquired. Sixteen samples were obtained before IVI of anti-VEGF antibody, whereas 30 samples were acquired after IVI of anti-VEGF antibody, with varying post-IVI times (Fig. 3). The basic characteristics of these 46 samples are shown in Table 2.

### VEGF concentrations before and after IVI

**Figure 4:**
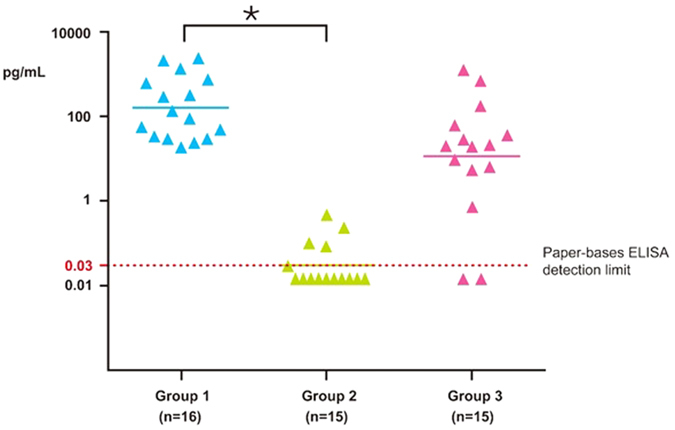
VEGF levels before and after IVI. Group 1: pre-IVI samples; group 2: samples collected within 5 weeks of IVI; group 3: samples collected more than 5 weeks after IVI.

The mean VEGF concentration in group 1 was 545.71 ± 810.29 pg/mL (mean ± SD, n = 16) before IVI of anti-VEGF antibody. In group 2, 66.67% of samples (10/15) achieved complete VEGF suppression (below the detection limit) within 5 weeks after IVI of anti-VEGF antibody; the mean VEGF concentration was 0.072 ± 0.131 pg/mL (Fig. 4). In group 3, only 13.33% of samples (2/15) achieved complete VEGF suppression at more than 5 weeks after IVI of anti-VEGF antibody, with a mean VEGF concentration of 163.06 ± 367.06 pg/mL (Table 3). The aqueous VEGF concentration was significantly lower in group 2 than in group 1 (*p *= 0.0143). Among the samples in group 3, 100% (10/10) were found to have elevated VEGF at more than 49 days after IVI of anti-VEGF antibody. One patient with PCV showed no VEGF suppression 3 weeks after IVI of anti-VEGF antibody (Fig. 3).

**Figure 5:**
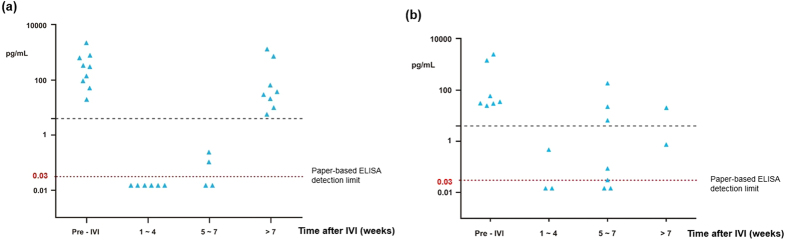
(**a)** Analysis of VEGF concentrations in patients receiving bevacizumab. (**b)** Analysis of VEGF concentrations in patients receiving ranibizumab.

Next, we evaluated the association between different IVI agents and VEGF suppression. Eyes in the ranibizumab group seemed to exhibit earlier VEGF elevation within 49 days after IVI (Fig. 5). In the bevacizumab group, 11.11% (2/18) of eyes were found to have earlier VEGF elevation within 49 days after IVI. Comparatively, in the ranibizumab group, 50.00% (6/12) of eyes were found to have earlier VEGF elevation within 49 days after IVI. The earlier VEGF elevation within 49 days after IVI was significantly associated with ranibizumab administration rather than bevacizumab administration (*p* = 0.0342).

### CFT and visual acuity before and after IVI

The CFT did not differ before and after IVI. However, the difference in CFT between groups 1 and 3 was nearly significant (*p* = 0.051). In contrast, visual acuity was significantly improved in group 2 (post-IVI within 5 weeks) compared with that in group 3 (post-IVI more than 5 weeks). Comparisons between other groups did not show significant differences.

## Discussion

IVI with anti-VEGF antibody is a widely applied therapy around the world. However, post-op conditions for evaluation primarily rely on optical imaging modalities, such as SD-OCT or FAG. However, SD-OCT only reflects structural changes in the retina, which appear later than true VEGF or other cytokine changes^9^,^30^. Second, SD-OCT can only yield qualitative findings, such as central foveal edema status, rather than biochemically based data, which is easier to quantify and monitor accurately. In this study, we established a P-ELISA method for application as a new POC diagnostic tool because it requires only 40 μL of sample to complete one set of semiquantitative results. Conventional ELISA requires dilution of samples, which reduces the sensitivity of the analysis. P-ELISA allows clinical ophthalmologists or researchers to evaluate VEGF levels with a simpler and more sensitive method. Luminex is another method for quantifying VEGF levels using only small samples of aqueous humor; however, the equipment required for this method limits its applications. P-ELISA for VEGF detection offers superior sensitivity compared with conventional ELISA or Luminex^19^,^20^. For example, our P-ELISA showed an additional five samples with no VEGF suppression compared with the results from conventional ELISA or Luminex. Additionally, the use of p-ELISA for quantification of VEGF in the aqueous humor has several advantages over conventional ELISA, including reduced sample volume, higher sensitivity, shorter assay times, and lower costs. To the best of our knowledge, this is the first study to report using a POC diagnostic tool, namely P-ELISA, to measure aqueous VEGF concentration before and after IVI of anti-VEGF antibody.

According to our literature review, the sensitivity of P-ELISA is greater than that for conventional ELISA and Luminex (0.03 pg/mL versus 4–5 pg/mL)^19^,^20^. Because P-ELISA requires no dilution step in its protocol, sensitivity is enhanced. Furthermore, the cost of Luminex is quite high, and the process is dependent on highly advanced equipment and technical training. Because this process can only be carried out in hospitals with adequate laboratories and trained personnel, its use in general healthcare by ophthalmologists is limited. Additionally, it is a considerable challenge to measure VEGF in aqueous humor samples owing to the small volume of aqueous samples that can be obtained, the relative impracticality of the method, and the high cost of the Luminex system.

In our study, 66.67% (10/15) of eyes in group 2 showed suppression of aqueous VEGF concentrations within 5 weeks after IVI with ranibizumab or bevacizumab. Only 13.33% (2/15) of eyes in group 3 exhibited complete VEGF suppression at more than 5 weeks after IVI of anti-VEGF antibody. Significant differences between eyes pre-IVI and post-IVI within 5 weeks and between the two post-IVI groups (groups 2 and 3) were also observed. Moreover, patients receiving IVI of ranibizumab tended to have earlier VEGF elevation. This result was reasonable because the vitreous half-life of intravitreal ranibizumab is shorter than that of bevacizumab^31^. These results were also consistent with typical clinical findings, in which not all patients have the same post-IVI response; accordingly, an “as needed” regimen is often recommended by many retinal specialists.

**Figure 6:**
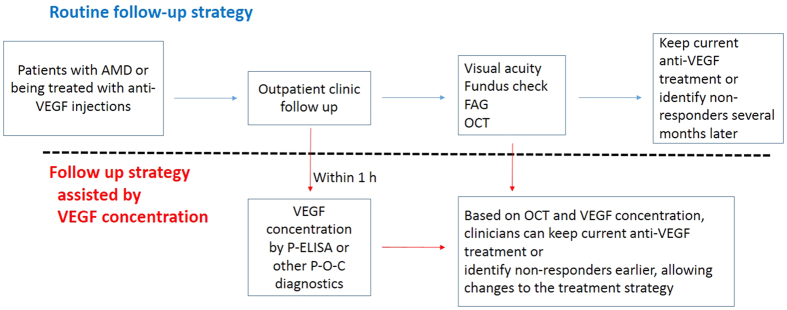
Schematic comparison of routine follow-up strategies and follow-up based on analysis of VEGF concentrations. In the routine strategy, most of the nonresponders can be identified after several anti-VEGF injections several months later, during which time devastating, irreversible vision loss can occur. Based on P-ELISA or other point-of-care (P-O-C) diagnostics, VEGF concentrations within the eye can provide the ophthalmologist with valuable information and facilitate decision-making regarding treatments options.

Every patient has a different rate of response to antibodies. Resistance to anti-VEGF treatment has also been reported and requires further clarifications^17^,^18^,^32^. For many of our patients, visual acuity worsened, even after receiving IVI anti-VEGF antibody, supporting that anti-VEGF antibody alone cannot stop retinal angiogenesis over a long period. Retinal angiogenesis is a multifactorial disease, and anti-VEGF cannot be applied in all patients with retinal angiogenesis. Thus, an individualized medical treatment plan should be considered based on different biochemical, SD-OCT, and FAG/ICG results. One report demonstrated that loss of intraocular VEGF suppression is always followed by morphological changes, as determined by SD-OCT, and loss of visual acuity is usually the last change^9^. Without prompt treatment to lower VEGF within the eye, a progressive cascade of damages occurs, increasing the likelihood of irreversible changes to visual acuity. Fortunately, the use of POC diagnostics that require only tiny sample volumes offers hope for close monitoring and early intervention. We recommend that nonresponders should be further classified through VEGF quantification and SD-OCT (e.g., high VEGF and persistent macular edema; Fig. 6). Anti-PDGF treatment, such as Fovista, has been shown to preserve visual acuity in phase 2 trials, and phase 3 trials are ongoing. Targeting both VEGF-A and PDGF receptor beta (PDGFRβ) enhance angiogenetic inhibition in mouse models of choroidal neovascularization and may be a breakthrough in the treatment of exudative AMD^33^. Furthermore, complement cascades may offer additional therapeutic opportunities; indeed, many drugs are available for this route, including C3 inhibitor (POT-4) and C5 inhibitor^34^.

As highlighted above, ophthalmologists are in need of novel diagnostic tools to monitor VEGF levels in patients with repetitive injection of IVI^30^. If VEGF levels are elevated earlier than presumed, retreatment or a shift to another anti-angiogenetic treatment is recommended. This assertion emphasizes the importance of POC diagnostics for VEGF or other cytokine detection, as it provides the ophthalmologist with immediate results and reduces the lag effect, thereby allowing earlier treatment.

There were several limitations to this study. First, the sample size was relatively small. Collection of more samples from patients having different disease spectra may yield more significant results in future analyses. For some patients, early VEGF elevation or lack of response could not be observed after IVI because of the lack of close longitudinal follow-up; moreover, it was impossible for patients to undergo aqueous humor sampling every week. As another limitation, we did not examine other cytokines, such as PDGF, related to angiogenesis. We suggest building an individualized prolife for every patient with longitudinal follow-up to monitor different cytokines for evaluation as biomarkers of response or resistance.

In conclusion, monthly IVI of anti-VEGF antibody yields durable VEGF inhibition. Elevation of VEGF was usually observed 5 weeks after IVI of anti-VEGF antibody. Earlier detection of VEGF elevation may help primary ophthalmologists decide whether to apply prompt retreatment or select another possible treatment regimen.

## Additional Information

**How to cite this article**: Hsu, M.-Y. *et al*. Detection of aqueous VEGF concentrations before and after intravitreal injection of anti-VEGF antibody using low-volume sampling paper-based ELISA. *Sci. Rep.* **6**, 34631; doi: 10.1038/srep34631 (2016).

## Figures and Tables

**Table 1:** Comparison between conventional and paper-based ELISA for aqueous VEGF quantification.

Equipment	P-ELISA for VEGF	Conventional ELISA for VEGF
	Desktop scanner and smartphone camera	Plate readout
Antigen/primary antibody	VEGF/HRP-conjugated avastin	VEGF/human recombinant VEGF-A antibody
Secondary antibody	None	HRP conjugate
Detection sensitivity	0.03 pg	18.75 pg/mL
Detection range	0.01–100,000 pg/mL	31.25–2000 pg/mL
Cost for equipment	100 USD	20000 USD
Dilution	No	Yes
**Reagent/duration**	**Volume (microliter)/time**	**Volume (microliter)/time**
(1) Immobilize VEGF	2/7 min	70/120 min
(2) Blocking	2/7 min	100/30 min
(3) Antibody	7.5/20 min	30/60 min
(4) Colorimetric reaction (add TMB+H_2_O_2_)	2/10 min	100/3 min
Total per zone	13.5/44 min	300/213 min
Total sample volume require per test	40 (repeat 20 wells)	9600 (total 96 wells)

**Table 2:** Basic parameters in subgroups.

	Group 1 (n = 16) Before IVI	Group 2 (n = 15) Post-IVI ≤ 5 weeks	Group 3 (n = 15) Post-IVI > 5 weeks	*P-value*		
				1 vs 2	1 vs 3	2 vs 3
Age (mean ± SD)	79.63 ± 7.88	75.33 ± 8.38	76.47 ± 10.35	0.15	0.34	0.74^a^
Female/male	4/12	6/9	4/11	0.46	1.00	0.70^b^
Latest IVI avastin/lucentis	not applicable	9/6	11/4	—	—	0.70^a^
AMD/myopic NV/PCV	15/0/1	12/2/1	14/0/1	0.33	1.00	0.60^a^
Follow-up months (mean ± SD)	14.38 ± 6.89	12.14 ± 6.67	18.00 ± 11.30	0.37	0.29	0.09^a^
No. of samples below detection limit of VEGF after IVI	0	9 (60%)	2 (13.3%)	—	—	< 0.05^*^^b^

^a^Kruskal-Wallis test.

^b^Fisher’s two-tailed test.

IVI = intravitreal injection, AMD = age-related macular degeneration, NV = neovascularization, PCV = polypoidal choroidal vasculopathy, VEGF = vascular endothelial growth factor, SD = standard deviation.

**Table 3:** Different parameters in the subgroups.

	Group 1 (n = 16) mean ± SD	Group 2 (n = 15) mean ± SD	Group 3 (n = 15) mean ± SD	*P-value*^a^		
				1 vs 2	1 vs 3	2 vs 3
VEGF (pg/mL)	545.71 ± 810.29	0.072 ± 0.131	163.06 ± 367.06	<0.05^*^	0.1049	0.096
CFT (μm)	439.57 ± 139.66	360.08 ± 199.34	318.43 ± 189.33	0.21	0.051	0.56
V.A.(log MAR)	1.82 ± 2.61	0.74 ± 0.30	1.18 ± 0.63	0.12	0.36	<0.05*

^a^Kruskal-Wallis test.

SD = standard deviation, VEGF = vascular endothelial growth factor, CFT = central foveal thickness (by SD-OCT), V.A. = visual acuity.
